# Novel candidate genes for lignin structure identified through genome-wide association study of naturally varying *Populus trichocarpa*

**DOI:** 10.3389/fpls.2023.1153113

**Published:** 2023-05-05

**Authors:** Nathan Bryant, Jin Zhang, Kai Feng, Mengjun Shu, Raphael Ployet, Jin-Gui Chen, Wellington Muchero, Chang Geun Yoo, Timothy J. Tschaplinski, Yunqiao Pu, Arthur J. Ragauskas

**Affiliations:** ^1^ Department of Chemical and Biomolecular Engineering, University of Tennessee, Knoxville, TN, United States; ^2^ Center for Bioenergy Innovation, Biosciences Division, Oak Ridge National Laboratory, Oak Ridge, TN, United States; ^3^ Department of Chemical Engineering, State University of New York College of Environmental Science and Forestry, Syracuse, NY, United States; ^4^ Center for Renewable Carbon, Department of Forestry, Wildlife, and Fisheries, University of Tennessee Institute of Agriculture, Knoxville, TN, United States

**Keywords:** genome-wide association studies (GWAS), *Populus*, nuclear magnetic resonance (NMR) analysis, lignin, p-hydroxybenzoate

## Abstract

*Populus* is a promising lignocellulosic feedstock for biofuels and bioproducts. However, the cell wall biopolymer lignin is a major barrier in conversion of biomass to biofuels. To investigate the variability and underlying genetic basis of the complex structure of lignin, a population of 409 three-year-old, naturally varying *Populus trichocarpa* genotypes were characterized by heteronuclear single quantum coherence (HSQC) nuclear magnetic resonance (NMR). A subsequent genome-wide association study (GWAS) was conducted using approximately 8.3 million single nucleotide polymorphisms (SNPs), which identified 756 genes that were significantly associated (−log_10_(*p*-value)>6) with at least one lignin phenotype. Several promising candidate genes were identified, many of which have not previously been reported to be associated with lignin or cell wall biosynthesis. These results provide a resource for gaining insights into the molecular mechanisms of lignin biosynthesis and new targets for future genetic improvement in poplar.

## 1 Introduction

Poplar (*Populus* sp.) is a promising lignocellulosic biomass feedstock due to its fast growth, ability to grow on marginal land, high cellulose content, and relatively low lignin content (Sannigrahi et al., 2010; Bryant et al., 2020). However, lignocellulosic biomass exhibits tremendous variability in cell wall traits, such as composition and structure. Cell wall structure and composition depend on factors such as environmental conditions (i.e., drought, cold stresses), biomass type (i.e., woody vs. herbaceous), tissue (i.e., stem vs. leaf), and genetic variations. One cell wall component that demonstrates a high degree of variability is lignin. Lignin is a complex and heterogeneous biopolymer that accounts for 18–32% (dry weight) of *Populus* (Sannigrahi et al., 2010; Li et al., 2016). It serves several important biological functions, including water transport, providing mechanical strength, and response to environmental stresses (Liu et al., 2018). However, since it acts as a natural barrier against pathogens, it is one of the main factors contributing to biomass recalcitrance to biological conversion to biofuels. Lignin is typically comprised of three primary monomers synthesized through the general phenylpropanoid and monolignol specific pathways: sinapyl alcohol, coniferyl alcohol, and *p*-coumaryl alcohol. When exported to the apoplast, these monolignols are oxidized by laccases and/or peroxidases, and then undergo radical cross-coupling reactions. Once coupled into the lignin polymer, these alcohols are identified as syringyl (S), guaiacyl (G), and *p*-hydroxyphenyl (H) units, respectively (Bose et al., 2009). It has also been shown that lignin demonstrates plasticity by incorporating non-canonical monolignols into the polymer, such as *p*-hydroxybenzoates (PB) (del Riío et al., 2020). These units are incorporated into the lignin polymer *via* a variety of interunit bonds, such as aryl ether (β-*O*-4), resinol (β-β), and phenylcoumaran (β-5), among others. The structure of lignin has potential bioenergy implications, as evidenced by the lignin S/G ratio association with biomass digestibility and conversion to biofuels (Li et al., 2016). Lignin structure can also influence lignin valorization. For instance, acetaminophen, the active ingredient in Tylenol, can be synthesized from the PB moiety (Ralph et al., 2019).

The formation of the secondary cell wall requires coordination of many metabolic pathways (Zhang et al., 2018a), presenting challenges in linking phenotypes to genetic mutations. Additionally, complex traits such as lignin are often controlled by several multigenic families. Genome-wide association studies (GWAS) are powerful tools for identifying polymorphic loci that contribute to phenotypic variation and sometimes trace-back to the genes or biological mechanisms involved. Due to the large sample size required for GWAS, high throughput techniques are typically utilized to analyze lignin traits. However, high throughput methods such as pyrolysis molecular beam mass spectroscopy (Py-MBMS) or near-infrared (NIR) spectroscopy provide limited information on lignin composition. These methods are generally used to estimate only the relative lignin to sugars ratio within the cell walls, or the S/G ratio for lignin itself. Genomic association mapping has been successfully employed on *Populus* for bioenergy traits including lignin content and S/G ratio (Porth et al., 2013; Fahrenkrog et al., 2017), biomass yield (Allwright et al., 2016), and cell wall sugars (Guerra et al., 2013). Consequently, the genetic basis of most lignin traits remains understudied by GWAS, and new methods for characterization of lignin phenotypes are urgently required. In comparison to Py-MBMS or NIR, the analytical technique heteronuclear single quantum coherence (HSQC) NMR, is more time and labor intensive, but has the potential to provide substantially more information on lignin structure.

In this study, we performed a deep phenotyping of the lignin polymer of 409 *P. trichocarpa* genotypes by HSQC NMR. By performing a detailed phenotyping of twelve lignin traits in a large population of poplar trees, we found that the lignin composition is highly variable across individuals, with evidence of incorporation of non-canonical monolignols into the polymer of lignin. Subsequently, a GWAS analysis enabled the identification of novel candidate genes that could explain the diversity in lignin composition. Most of the candidate genes identified were not previously reported to be associated with lignin or cell wall biosynthesis. This provides a resource for gaining insights into the molecular mechanisms of lignin biosynthesis and new targets for future genetic improvement in poplar.

## 2 Materials and methods

### 2.1 Biomass preparation

Wood samples for this study were collected from three-year-old *Populus trichocarpa* grown in a common garden in Corvallis, OR (44°34′14.81″N 123°16′33.59″W). Site establishment and management practices were previously described by Muchero et al. (Muchero et al., 2015). One-centimeter-diameter increment cores were collected at breast height for the 409 genotypes in January 2013. Cores were stored in zip-lock bags at −20°C before processing. Wood cores were air dried at room temperature before they were ground using Wiley Mini-Mills (Swedesboro, NJ) with a 20-mesh screen. Ground samples were stored in glass vials at room temperature.

### 2.2 Lignin sample preparation

Each sample was Soxhlet extracted using toluene/ethanol (2:1, *v:v*) for at least eight hours and subsequently air-dried for at 24 hours. Approximately 500 mg of each extractives-free sample was ball-milled for two hours at 600 RPM (at five-minute intervals) on a Retsch planetary ball mill. The ball-milled biomass was then subjected to enzymatic hydrolysis with cellulase (Sigma-Aldrich) in a sodium acetate buffer at 37°C and 200 RPM for 48 hours. The solid lignin enriched residues were then separated *via* centrifugation and lyophilized for 48 hours for NMR analysis.

### 2.3 NMR analysis

Lignin structure was analyzed by 2D HSQC NMR with a Bruker Avance II 500-MHz spectrometer. Approximately 40 mg of lignin enriched residue was dissolved in 0.5 mL of DMSO-*d*_6_ in a 5 mm NMR tube and sonicated for one hour. The Bruker pulse sequence hsqcetgpsip2.2 was utilized on a N_2_ cryoprobe with the following parameters: spectra width of 12 ppm in the ^1^H dimension with 1024 data points; spectra width of 220 ppm in the ^13^C dimension with 256 increments and 32 scans. The HSQC spectra were analyzed with Bruker TopSpin 3.5pl6 software. The DMSO-*d*_6_ solvent peak (δ_C_/δ_H_ at 39.5/2.49) was used to calibrate the chemical shifts. At least annually, the repeatability and experimental error of the HSQC measurement are quantified by analyzing a standard *Populus* sample. Lignin was isolated from the standard *Populus* sample per the method described above, and three separate samples were analyzed by the same pulse sequence. For this most recent analysis, the standard deviation of the three samples ranged from 0.1 (for H unit) to 1.4 (β-*O*-4 linkage). The coefficient of variation (CV) may be considered a better measurement of variability, since it is defined as the ratio of the standard deviation to the mean expressed as a percentage. The CVs of the standard *Populus* samples ranged from 1.4% (S unit) to 18.9% (β-5 linkage). A study utilizing ^1^H NMR for biomarker analysis identified that larger peaks exhibited a CV of 5–10%, whereas smaller peaks had CV in the 15–30% range (Wang et al., 2013), which is consistent with our observed measurements.

### 2.4 Genome-wide association study

Whole genome resequencing, single nucleotide polymorphism (SNPs)/nucleotide insertions and deletions (indels) calling and SnpEff analysis for the 917 individuals of this *Populus* GWAS population was previously described by Zhang et al. (2018b). The *P. trichocarpa* Nisqually-1 reference genome v3.1 was used for read alignment and variant calling. The resulting SNP and indel dataset is available at http://bioenergycenter.org/besc/gwas/. This study utilized genotypic data for a subset of 409 genotypes from this dataset. To assess genetic control, we used the GEMMA software to calculate kinship for the *Populus* GWAS population as the correction factor for genetic background effects (Zhang et al., 2018b). Genotype-to-phenotype associations were performed using 8,301,860 SNP and indel variants with minor allele frequencies > 0.05. The HSQC spectra from 2D HSQC NMR were used as phenotypes. Statistical significance of associations was evaluated using the Storey’s Q-value threshold. Deviation of *p*-values from normality was assessed using quantile-quantile (Q-Q) plots. Candidate gene identification and RNAseq mapping for co-expression analysis were performed using the *P. trichocarpa* v3.1 reference genome. RNAseq of xylem tissue of 378 *Populus trichocarpa* transgenics plants knockdown or overexpressing monolignol genes and transcription factors involved in the regulation of cell wall biosynthesis were downloaded from the Sequence Read Achieve (SRA; accession: PRJNA314500) (Matthews et al., 2021). Library quality was assessed using FastQC (v0.11.9; https://www.bioinformatics.babraham.ac.uk/projects/fastqc/), residual adapters and low-quality reads were trimmed using Trimmomatic v0.39 (Bolger et al., 2014) reads were mapped to the reference genome using STAR v 2.7.6a (default parameters and –outFilterMultimapNmax 100 (Dobin et al., 2013) and transcript per million (TPM) values were extracted for all annotated genes using Stringtie (Pertea et al., 2015) 18 samples with low mapping rates (<80% of mapped reads) were excluded for the subsequent analysis. Co-expression of candidate genes with 86 phenylpropanoids and lignin-related genes (**Table SI 3**) was estimated by calculating pairwise Pearson correlation coefficient (PCC) across 360 samples using the function rcorr() from the Hmisc R package (Shi et al., 2010; Sundell et al., 2017). For each potential candidate gene, multiple individual scores were calculated: (a) significance threshold of −log_10_(*p*-value)=6, 7, and 8 were assigned an individual score of 1, 2, and 3, respectively; (b) connectivity with SNPs was scored according to log_10_(number of connected SNPs); (c) connectivity of the SNPs with phenotypes; number and average value of significant co-expression associations (|PCC| ≥ 0.5, FDR < 0.001) with lignin-related genes. All individual scores were scaled to obtain values ranging from 0 to 1. These individual scores were summed to obtain a final overall score, which was utilized to prioritize candidate genes for consideration (higher scoring genes were considered best candidates).

## 3 Results

### 3.1 Lignin chemistry

**Figure 1:**
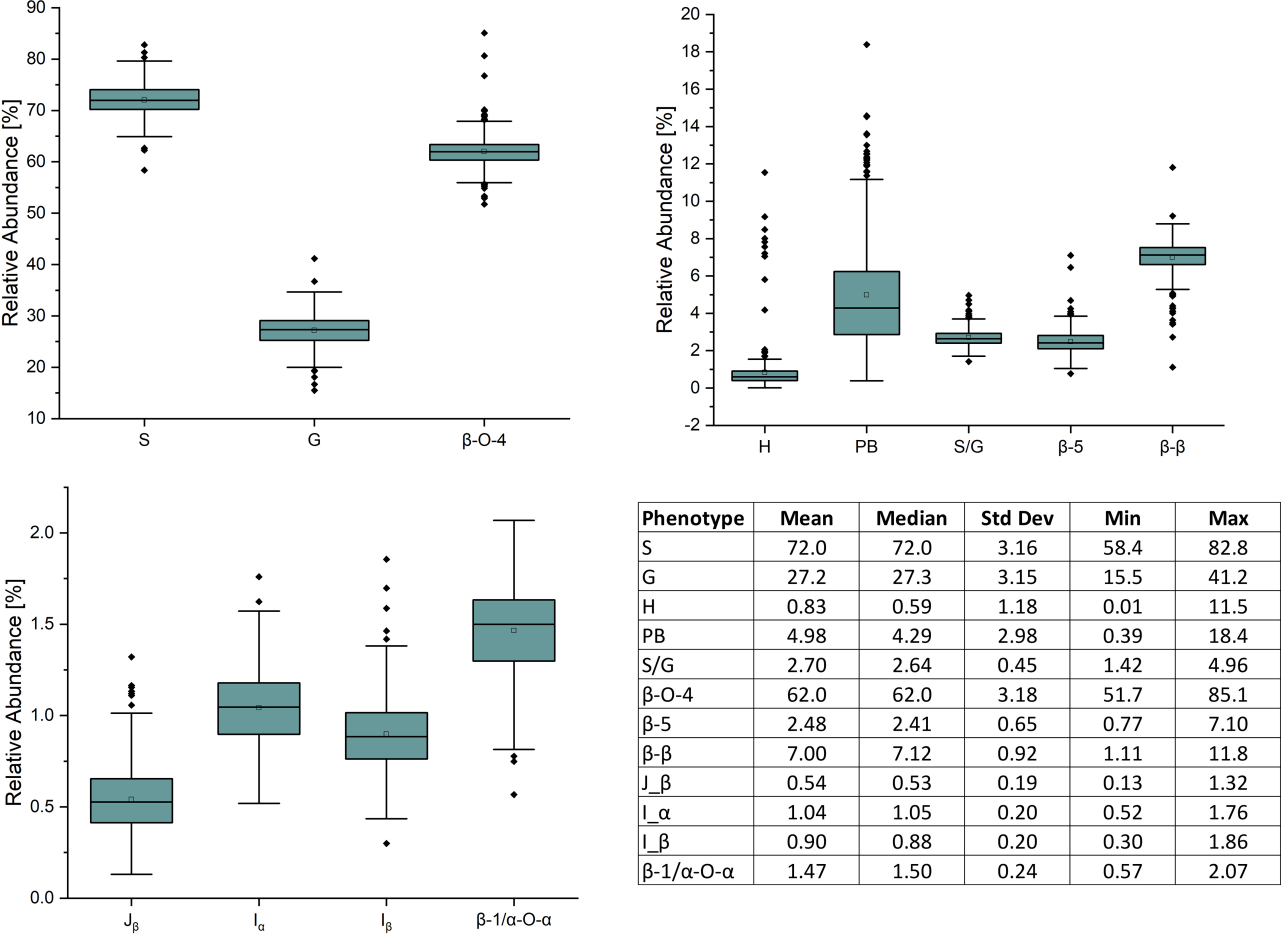
Box plot diagrams for the distributions of lignin phenotypes as measured by HSQC NMR – [Top left] syringyl (S), guaiacyl (G), and aryl ether linkages (β-O-4); [Top right] p-hydroxypehnyl (H), p-hydroxybenzoate (PB), syringyl:guaiacyl ratio (S/G), resinol linkages(β-β), and phenylcoumaran linkages (β-5); [Bottom left] cinnamyl aldehyde end groups (J_β_), cinnamyl alcohol end groups (I_α_/I_β_), and spirodienone linkages (β-1/α-O-α). Bottom right: descriptive population statistics of each lignin phenotype.

**Figure 2:**
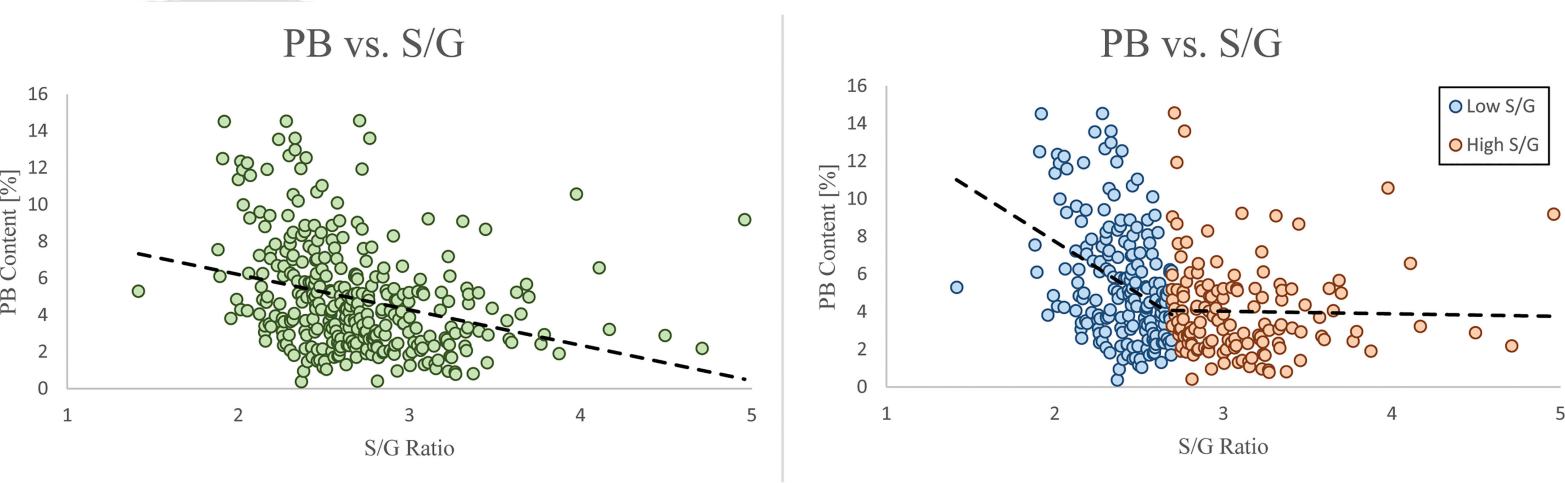
The PB and S/G ratio phenotypes have an overall negative correlation (left). This negative correlation is significantly stronger when the S/G ratio < 2.7, and significantly weaker when the S/G ratio is >2.7 (right).

409 unique poplar genotypes were analyzed by HSQC NMR to elucidate structural information for twelve lignin phenotypes, including S units, G units, H unit, PB units, S/G ratio, cinnamyl alcohol end groups (I_α_, I_β_), cinnamyl aldehyde end groups (J_β_), β-O-4 aryl ether linkages, β-5 phenylcoumaran linkages, β-β resinol linkages, and β-1/α-O-α spirodienone linkages. with results summarized in **Figure 1**. As expected, the lignin of the *P. trichocarpa* population is comprised primarily of S and G units, with an average of approximately 72.0 and 27.2 units per 100 S+G+H units, respectively. As expected, H units were the least abundant monolignol, averaging 0.8 units per 100 S+G+H units. The quantity of S units was measured to be as low as 58.4% and as high as 82.8%. Similarly, G units ranged from 15.5% to 41.2%. This resulted in a population average S/G of 2.70, though values ranged from 1.42 to 4.96. The next most abundant phenotype was PB. While PB content averaged 4.87%, levels across the population varied significantly ranging from near background (0.39%) to a non-trivial 18.4% – approximately the lower limit of G unit content. However, within ±0.1 of the average S/G ratio (i.e., 2.60–2.80), PB content ranges from near background levels (1.33%) to 14.6%. To further explore this relationship, the samples were subdivided into a low S/G ratio fraction (<2.70) and a high S/G fraction (>2.70), as shown in **Figure 2** (right). In the low S/G ratio sample fraction, the correlation between PB and S/G ratio remains statistically significant, with an average PB content of 5.5%. However, in the high S/G ratio sample fraction, this correlation does not hold, and the average PB content is slightly lower at 4.0%. Additionally, 21 of 24 high PB outliers (i.e., PB >11.3%) appeared in the lower range of S/G ratios, with no high PB outlier coming from a sample with an S/G ratio above 2.77. It was confirmed that the H unit constituted only a minor fraction of the lignin polymer, averaging just 0.91%. However, like PB, H unit levels were highly variable, ranging from nondetectable to as high as 11.5%. The β-O-4 aryl ether linkage was by far the most abundant interunit linkage, averaging approximately 62 per 100 aromatic units. It was also the most variable linkage, measuring as high as 85.1% and as low as 51.7%. The β-β resinol linkages were shown to make up a minority of interunit linkages, averaging 7 per 100 aromatic units, with an upper limit of 11.8% and a lower limit of 1.11%. On the other hand, β-5 phenylcoumaran, which averaged 2.48%, ranged from as low as 0.77% to as high as 7.10%. The spirodienone (β-1/α-O-α) linkage was present in small but detectable quantities, averaging 1.47%, but not exceeding 2.07%. The population statistics for these phenotypes are summarized in **Table 1**. The data is also displayed in box plot form in **Figure 1** for a visual comparison. Altogether, the large variations observed in both the subunit content and the type linkages, suggest that a number of polymorphisms segregating within the population can drastically affect the structure of the polymer of lignin.

**Table 1:** Summary of a select subset of candidate genes from GWAS results.

Phenotype	Gene ID	*p*-value	Connectivity with SNPs	Average co-expression with phenylpropanoid genes	Annotation (Arabidopsis alias)
H	Potri.006G169600	8.89E-11	17	0.63	4-coumarate:CoA ligase 2 (4CL2)
H	Potri.001G045100	3.63E-08	2	0.62	cinnamoyl CoA reductase 1 (CCR1)
β-5	Potri.003G059200	8.74E-07	28	0.67	lysophospholipase 2 (CSE)
S	Potri.008G155500	1.16E-07	16	0.58	20S proteasome beta subunit D1 (PBD1)
G		3.69E-09			
S/G		5.46E-08			
S	Potri.006G176600	1.06E-09	27	–	XB3 ortholog 2 in *Arabidopsis thaliana* (XBAT32)
G		7.88E-09			
S/G		4.52E-07			
PB	Potri.T017000	7.88E-08	1	0.58	glutamine dumper 1 (GDU1)
PB	Potri.T017100	7.88E-08	1	–	glutamine dumper 2 (GDU2)
β-β	Potri.004G077700	7.75E-08	4	0.64	P-loop containing nucleoside triphosphate hydrolases superfamily protein
β-O-4		1.11E-24			
H	Potri.015G082700	3.32E-08	1	0.66	PtrMYB074 (AtMYB50)
β-β	Potri.019G040900	3.72E-08	4	0.57	MYB domain protein 105
PB	Potri.004G073900	7.97E-07	1	–	Pectin lyase-like superfamily protein
PB	Potri.008G099300	8.78E-07	1	0.66	S-adenosylmethionine synthetase family protein (MAT4)
H	Potri.010G064000	1.51E-07	4	0.59	MYB domain protein 79
β-O-4	Potri.004G174200	5.18E-08	2	0.61	proteasome alpha subunit D2 (PAD2)
β-O-4	Potri.008G011100	1.99E-07	1	–	Plant invertase/pectin methylesterase inhibitor superfamily
PB	Potri.014G142000	3.75E-07	1	0.51	galacturonosyltransferase 15 (GAUT15)
I_β_	Potri.005G163900	1.91E-07	9	–	S-adenosyl-L-methionine-dependent methyltransferases superfamily protein (OSU1)

The full list of genes identified by GWAS can be found in the Supplementary Information.

Genes are selected based on criteria such as putative cell wall or lignin biosynthesis function, strength of association, connectivity with SNPs, and co-expression with phenylpropanoid genes.

### 3.2 Genome-wide association study (GWAS) of phenotypic variation

**Figure 3:**
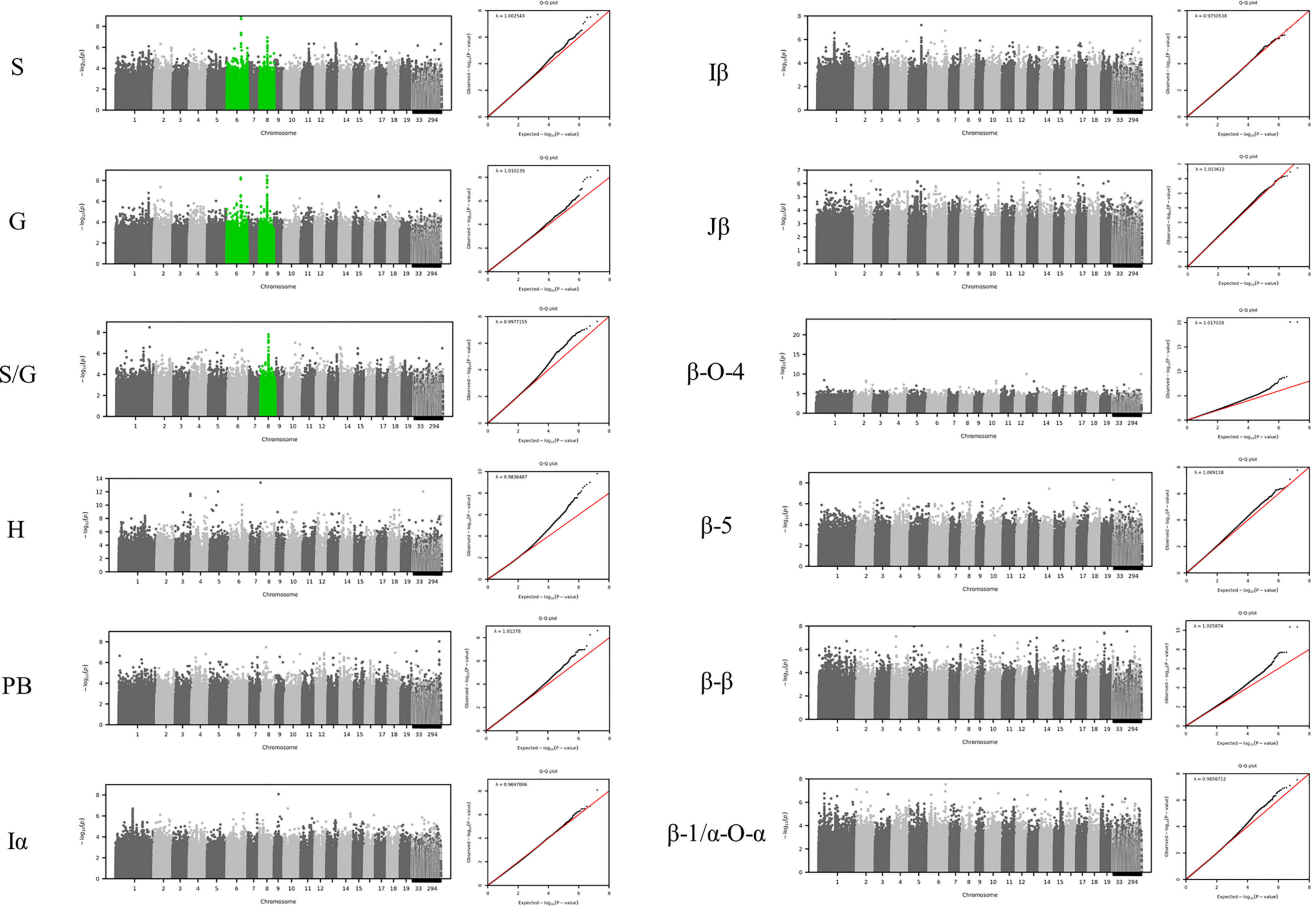
GWAS Manhattan plots below display correlations between SNPs and the specified lignin phenotype. Chromosome 8 is highlighted green for the S, G, and S/G phenotypes to indicate a significant association of 20S proteasome beta subunit D1 (PBD1) was observed for each of these phenotypes. Chromosome 6 is highlighted green for the S and G phenotypes to indicate that XB3 ortholog 2 (XBAT32) was significantly associated with these phenotypes. However, Chromosome 6 is not highlighted green for the S/G phenotype to indicate a lack of significant association of XBAT32 with this phenotype.

To identify the genomic loci controlling lignin phenotypes described above, we performed a GWAS using 409 unrelated *P. trichocarpa* accessions that had genotypic information represented in a panel of > 8.3 million single nucleotide polymorphisms (SNPs) and nucleotide insertions and deletions (indels) as described in the materials and methods. Associations with the phenotypes β-O-4, β-β, β-5, β-1/α-O-α, S, G, H, I, PB, and S/G ratio were tested in this analysis. The GWAS was conducted at increasing significance thresholds (i.e, −log_10_(*p*-value)=6, 7, & 8) to differentiate the strength of the associations. At the threshold of −log_10_(*p*-value)>6, the GWAS identified 756 genes that were significantly associated with at least one phenotype. The Manhattan plots and associated QQ plots for each lignin phenotype are displayed in **Figure 3**.

At the lowest threshold (−log_10_(*p*-value)=6), a total of 907 unique genes were detected in the vicinity or overlapping with the significant SNPs associated with at least one phenotype. A gene ontology (GO) analysis was performed to detect any enrichment in specific biological function among these candidate genes. A significant enrichment in cell wall related GO terms was detected for the highest significance thresholds (−log_10_(*p*-value)=7 and −log_10_(*p*-value)=8) (**Supplementary Figure SI 4**). The enrichment in cell wall related genes (relative to total number of genes) identified at each of these levels were 1.52, 1.87, and 2.48, respectively, when considering Poplar gene annotations. When considering Arabidopsis gene annotations, a similar trend was observed with the enrichment increasing from 2.70 to 5.79 between the −log_10_(*p*-value) = 6 and −log_10_(*p*-value) = 8 thresholds. The increase in enrichment for specific GO terms for higher GWAS significance thresholds provides evidence that the approach is successful at capturing the most likely causal genes for phenotyping variations in lignin composition, as opposed to a simple random sampling of genes from the genome. At the −log_10_(*p*-value)=6 threshold, 32 of the 756 genes identified by GWAS have previously been associated with cell wall biosynthesis in *Populus* or *Arabidopsis*. The GWAS identified several genes (Potri.006G169600, Potri.001G045100 and Potri.003G059200), which are predicted to encode key enzymes of the phenylpropanoids pathway: 4-coumarate:CoA ligase 2 (4CL2), cinnamoyl CoA reductase 1 (CCR1), and caffeoyl shikimate esterase (CSE), respectively. In addition, possible homologs of AtTRA2 (Potri.003G161900) and AtMAT4 (Potri.008G099300) were found associated with H-lignin and PB content (**Table SI 4**). In previous studies, perturbations in these two genes induced drastic changes in lignin content and structure in Arabidopsis (Shen et al., 2002; de Vries et al., 2018). In addition to effector genes directly involved in metabolic pathways, the GWAS highlighted higher level regulators as potential candidates, including major transcriptional regulators. PtrMYB074 (Potri.015G082700), strongly associated with H-lignin content in our GWAS, is a master regulator of secondary cell wall formation in poplar, and was shown to directly regulate the biosynthesis of lignin in wood forming tissues of mutant *P. trichocarpa* (Chen et al., 2019). Potri.001G346600 encoding a possible homolog of AtMYB21 was also found strongly associated with H-lignin content. In Arabidopsis AtMYB21 promotes flavonol biosynthesis through the regulation of FLS1, and was related to stress response and hormonal signaling (Zhang et al., 2021).

Interestingly, numerous candidate genes highlighted by this approach are predicted to be involved in the biosynthesis of cell wall polysaccharides, such as Potri.002G135500, a possible homolog of ATRAB6A involved in cellulose biosynthesis (He et al., 2018). Additionally, Potri.013G082200 and Potri.003G074600 are predicted to be a cellulose synthase (AtCSLD3) and a pectin lyase (AtQRT3), respectively. This GWAS approach also identified a significant association with Potri.001G248700, the closest putative ortholog of the *Arabidopsis* LACCASE 4, shown to be directly involved in lignin polymerization (Berthet et al., 2011).

**Figure 4:**
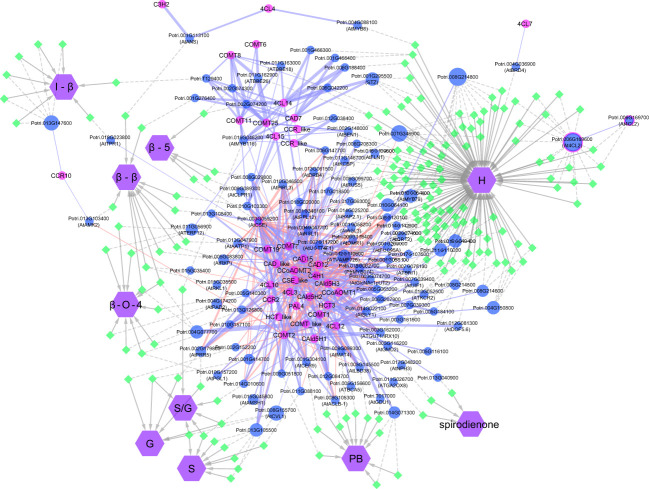
Visual representation of the network analysis connecting phenotypes, SNPs, flanking genes, and phenylpropanoid/lignin biosynthesis genes. Genes involved in lignin biosynthesis in *Populus* are named according to Shi et al. (2010). For candidate genes co-expressed with lignin biosynthesis genes, the *Populus* accession number is provided, with the gene alias of the closest *Arabidopsis* match in brackets.

The GWAS pointed to several other genes (**Figure SI 4**; **Table SI4**) which have not been previously associated with lignin or cell wall biosynthesis. To highlight top candidates, all genes were scored based on criteria such as strength of association (*p*-value of nearby SNPs), connectivity with SNPs, and co-expression with phenylpropanoid genes computed from external datasets (**Figure 4**; **Supplemental Table 1**). One of the highest scored candidate genes was Potri.008G155500, which is annotated as 20S proteasome beta subunit D1 (PBD1). This candidate gene was highly associated with 16 SNPs across the S, G, and S/G phenotypes (**Figure SI 4**). Another highly scored candidate gene was Potri.006G176600, which is annotated as XBAT32. The XBAT32 associations are peculiar in that this gene exhibits strong associations with the S and G phenotypes, but is not strongly associated with the S/G ratio, as presented in the Manhattan plots in **Supplementary Figure SI 4**. Together with major regulators such as PtrMYB074, and possible homologs of key biosynthetic enzymes such as AtMAT4 and AtTRA2, as mentioned previously, other candidate genes were found strongly co-expressed with lignin biosynthesis genes across transcriptomes of lignin perturbed poplar transgenic plants (**Figure 4**) (Wang et al., 2018). Notably, this approach highlighted candidates for the formation of PB. Glutamine dumper 1 (GDU1; Potri.T017000), associated with the PB phenotype, was found to be co-expressed with CAld5H3, a key gene in the lignin biosynthesis pathway that is known to influence S/G ratio (**Figure 4**). Among the other candidates associated with the PB phenotype, Potri.005G145500 was found negatively co-expressed with PtrCOMT2 and two CSE-encoding genes, involved in lignin biosynthesis in poplar. Potri.005G145500 is a potential ortholog of a group of LBD transcription factors AtLBD37/38/39 that were shown to regulate anthocyanin biosynthesis in Arabidopsis (Rubin et al., 2009). The candidate genes reported here, as well as other potential candidate genes of interest, are summarized in **Table 1**. All together, these results demonstrate that the GWAS performed highlighted multiple classes of candidate genes for the biosynthesis of the different moieties and linkages that constitute the lignin polymer.

## 4 Discussion

An advantage of analyzing 409 P*. trichocarpa* samples by HSQC is that the variation of lignin phenotypes, even those difficult to elucidate by other analytical methods or present at low levels, across the entire population can be better understood. The population statistics of the PB phenotype from this GWAS population was unique as it was the only phenotype which did not conform to a normal (or approximately normal) distribution. *Populus* is one of the few species which contain PB – a free phenolic pendant unit which has been shown to be conjugated to the γ-position of syringyl units (Stewart et al., 2009; Ralph et al., 2012). The abundance of PB could be of special interest to biorefinery considerations, either by utilizing PB derivatives as value-added products (Ralph et al., 2019) or as a method of improving biomass deconstruction by increasing the number of ester-linked phenolics (Sibout et al., 2016). While the biological function and biosynthesis remain largely unknown, recent work has identified an acyltransferase (Potri.001G44800) which was shown to control *p*-hydroxybenzoylation (de Vries et al., 2021; Zhao et al., 2021). This acyltransferase is co-expressed with lignin biosynthesis genes ferulate 5-hydroxylase/coniferaldehyde 5-hydroxylase (F5H/CAld5H), which have been shown to influence lignin S/G ratio (Baucher et al., 1999; Al-Haddad et al., 2009), which would support an observed relationship between PB and S/G ratio. However, these studies observed changes in PB content without an associated impact on S/G ratio (and other lignin phenotypes), demonstrating that the two can be delineated. Further understanding of PB biosynthesis and its relationship with S/G ratio could provide valuable insight towards engineering lignin for biorefinery applications. For the 409 genotypes considered in this study, PB is negatively correlated with S/G ratio (**Figure 2**, left), which is consistent with previous observations (Stewart et al., 2009; Yoo et al., 2018). More recently, a similar negative correlation was observed in a collection of 316 P*. trichocarpa* analyzed by saponification and HPLC (Mottiar & Mansfield, 2022). It has previously been hypothesized that PB (and other acylated units) are produced as a method of promoting syringyl-rich lignin. However, a negative correlation between PB and S units suggests this is not the case. Indeed, PB is a non-canonical monolignol that remains poorly understood. Therefore, GWAS associations related to PB may be considered especially interesting and shed new light underlying the genetic mechanisms underlying PB biosynthesis. As previously mentioned, one of the highest scored candidate genes associated with PB is GDU1. This is due, in part, to its co-expression to CAld5H3, which is known to catalyze a key reaction step in the lignin biosynthesis pathway and therefore heavily influence the S/G ratio. Because there is a clear correlation between PB and S/G, one may attribute this to being an artifact of CAld5H3 expression, rather than GDU1. However, manipulation of CAld5H3 in rice did not impact p-coumarate (the analogous PB ester in grasses) (Takeda et al., 2017). It has previously been shown that GDU1 is localized at the plasma membrane and is involved with nonselective amino acid export from plant cells into the apoplast (Réjane Pratelli et al., 2012). Export of amino acids (including phenylalanine) increased when GDU genes were overexpressed in Arabidopsis (Pratelli et al., 2009). However, the transport mechanism(s) by which lignin monomers are transferred from the cytosol to the apoplast remain unresolved (Perkins et al., 2019). Additionally, the association of glutamine synthesis with ammonia removal following phenylalanine and tyrosine conversion to *trans*-cinnamic acid and *p*-coumaric acid, respectively, early in the lignin pathway, has recently been reported in *Brachypodium distachyon* (Barros et al., 2022). Given such, GDU1 may play a role in preventing excessive accumulation of glutamine associated with the synthesis of lignin precursors. As another point of emphasis, glutamine dumper 2 (Potri.T017100) was also associated with the PB phenotype. Based on these factors, GDU1 could be further evaluated for potential impacts of PB incorporation into the lignin polymer.

The S/G ratio is perhaps the most well-studied lignin trait. Yet, the GWAS analyses identified strong associations with several genes which have not been previously linked to lignin biosynthesis. This is not entirely surprising, as the formation of the secondary cell wall requires coordination of many metabolic pathways (Zhang et al., 2018a), and as previously mentioned, one highly associated candidate gene is the 20S proteasome gene PBD1. This candidate gene, located on chromosome 8, is strongly associated with the S, G, and S/G ratio. The 20S proteasome has been shown to degrade client proteins to amino-acid residues (Tanaka, 2009). It is also the proteolytic core of the 26S proteasome, which mediates proteolysis and plays a key role in the regulation of critical cellular processes, such as transcriptional control, cell cycle progression, and stress response. Staszczak et al. (Staszczak, 2007), using *in vivo* blocking of proteasome function, indicated that the proteasomal pathway is involved in the regulation of activity of some ligninolytic enzymes (such as laccase) under nutrient deprivation in lignin-degrading Basidiomycete *Phlebia radiata*. The 26S proteasome pathway has been implicated in aspects of secondary cell wall biosynthesis, such as in cotton fiber development (Feng et al., 2018). Transgenic lines exhibited differences in expression of both cellulose and lignin biosynthesis genes, resulting in increased levels of lignin or lignin-like phenolics (Feng et al., 2018). Another strong association identified by the GWAS analysis was XBAT32. The association of XBAT32 is interesting, as it was observed to be associated with the S and G phenotypes independently, but was not observed to be associated with the S/G ratio. This is quite peculiar since the S/G ratio is quite literally the quotient of the S and G unit measurements. Of the three primary monolignols found in poplar, H units are typically present in low abundance (approximately 1%), and as a result, the ratio of S and G units tend to vary proportionally. XBAT32 was initially identified as a regulator of lateral root development in *Arabidopsis* plants with an XBAT32 mutation (Nodzon et al., 2004). It was later shown that XBAT 32 mutants produced increased levels of ethylene (Prasad et al., 2010) and therefore, by extension, plays a role in abiotic stresses response (Prasad & Stone, 2010). Another XBAT protein, XBAT35, has also been shown to be involved in ethylene signaling (Carvalho et al., 2012). A similar gene identified in cotton, GhXB32A, was also shown to function in response to stress (Ge et al., 2021). It is well-documented that stress can induce changes to lignin properties (Moura et al., 2010). It is therefore quite reasonable to implicate the stress related XBAT32 gene could be influencing lignin structure.

In conclusion, the lignin of 409 unique, natural variant *P. trichocarpa* genotypes were analyzed by HSQC NMR. A subsequent GWAS analysis identified 756 SNPs significantly associated among the twelve lignin phenotypes. The GWAS results include putative lignin and cell wall biosynthesis related genes. Subsequent gene ontology analyses show that cell wall related term enrichment increases with GWAS significance levels. These results provide evidence that the GWAS analyses identified causal genes, rather than randomly sampling the genome. Several candidate genes not previously associated with lignin or cell wall biosynthesis were identified by GWAS, including GDU1, PBD1, and XBAT32. These GWAS results can be used as targets for future work investigating lignin structure, and the functional characterization of these genes may reveal novel genetic mechanisms controlling lignin biosynthesis.

## Data availability statement

The datasets presented in this study can be found in online repositories. The names of the repository/repositories and accession number(s) can be found in the article/**Supplementary Material**.

## Author contributions

J-GC, WM, and AR conceived and designed the study. NB, CY, and YP characterized lignin structure. JZ, KF, MS, RP, and WM conducted the GWAS, GO enrichment, and network analyses. NB, JZ, KF, MS, RP, TT, and WM wrote the manuscript. All authors contributed to the article and approved the submitted version.
