# Supplementary material for: Novel candidate genes for lignin structure identified through genome-wide association study of naturally varying Populus trichocarpa
Source: Front Plant Sci. 2023 May 5;14:1153113. doi: 10.3389/fpls.2023.1153113 (PMC10197963; doi:10.3389/fpls.2023.1153113)
Supplement: Supplementary file 1 [file DataSheet_1.docx]

Supplementary Information for:

**Novel Candidate Genes for Lignin Structure Identified Through Genome-Wide Association Study of Naturally Varying *Populus* *trichocarpa***

Nathan Bryant^a^, Jin Zhang^b^, Kai Feng^b^, Mengjun Shu^b^, Raphael Ployet^b^, Jin-Gui Chen^b^, Wellington Muchero^b^, Chang Geun Yoo^c^, Timothy J. Tschaplinski^b^, Yunqiao Pu^b^, Arthur J. Ragauskas^a,b,d^

^a^ Department of Chemical and Biomolecular Engineering, University of Tennessee, Knoxville, Tennessee 37996, United States

^b^ BioEnergy Science Center & Center for Bioenergy Innovation, Biosciences Division, Oak Ridge National Laboratory, Oak Ridge, TN 37831, USA

^c^ Department of Chemical Engineering, State University of New York College of Environmental Science and Forestry, Syracuse, NY 13210 (USA)

^d^ Department of Chemical and Biomolecular Engineering, University of Tennessee, Center for Renewable Carbon, Department of Forestry, Wildlife, and Fisheries, University of Tennessee Institute of Agriculture, Knoxville, TN 37996 (USA)

Corresponding author: Arthur J. Ragauskas ([aragausk@utk.edu](mailto:aragausk@utk.edu))

**Table SI 1 –** **Abbreviations of lignin phenotypes**

**Figure SI 1 - Population distribution of lignin phenotypes**


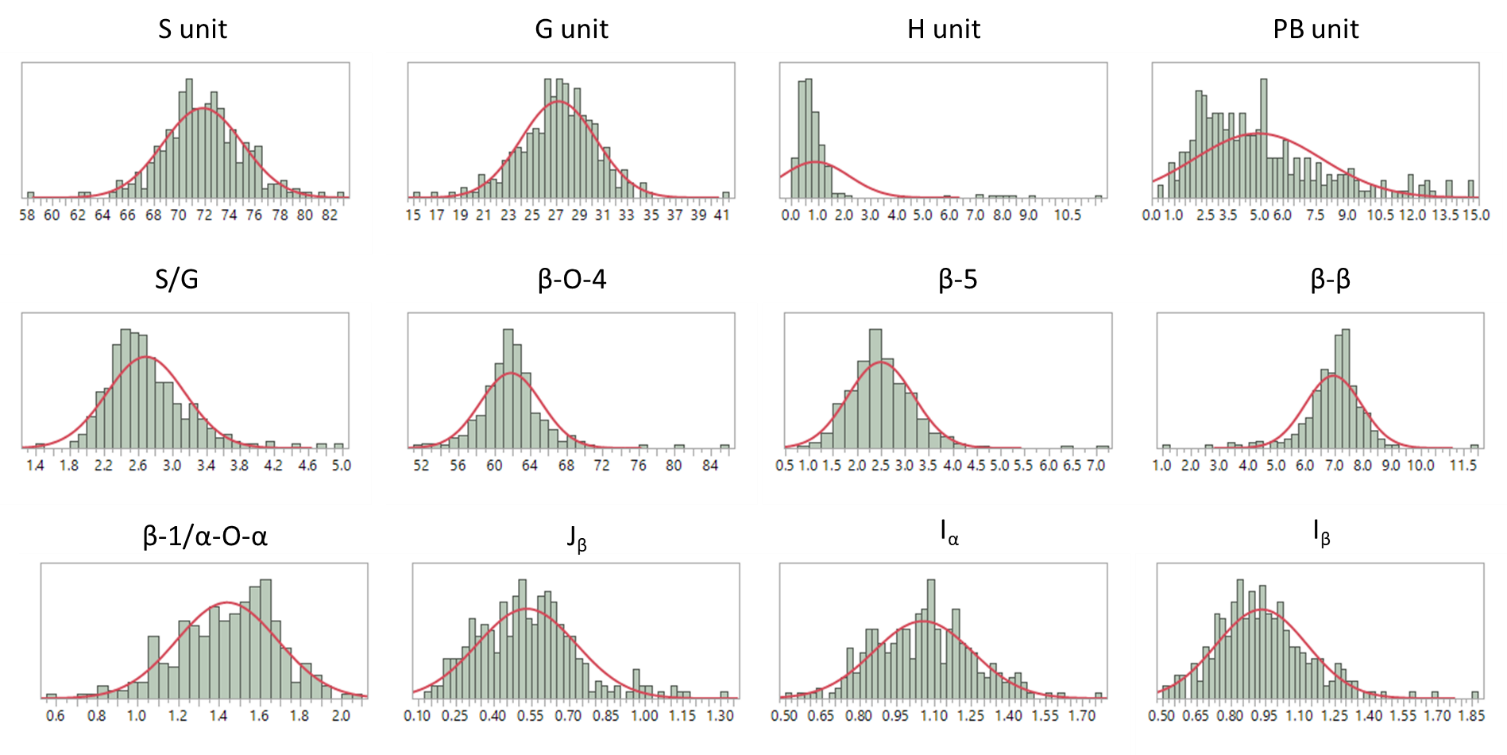


Figure SI1 – Population distributions of twelve lignin structural phenotypes quantified by HSQC NMR. Each distribution has been fitted with a normal distribution curve (red). All phenotype distributions, with the exception of PB, were found to be normal or approximately normal. PB was found to conform to a lognormal distribution. The H unit distribution is modeled including the 10 high outliers described in the main text.

**Figure SI 2 - Protease treatment of high H unit samples**

There were 10 samples from the GWAS population that were measured to have high levels of H units and identified as outliers by JMP statistical software. It has been reported that amino acid contamination can potentially contaminate the H_2/6_ signal in the HSQC spectra, resulting in the overestimation of H units. To test for potential contamination, four high H unit samples were selected for protease treatment. Protease treatment was conducted per a published procedure (Kim et al., 2017). Briefly, enzyme lignin (EL) was combined with Proteinase K in a phosphate buffer and shaken/incubated for 48h. Afterwards, samples were washed twice with DI water and lyophilized. Samples were then prepared and analyzed via HSQC NMR per the procedure in the main text. The abundance of H units in the enzyme lignin (before protease) and the protease treated lignin (after protease) are summarized in Figure SI2 below. The protease treatment reduced H unit measurements by HSQC an average of 32%. The largest reduction was observed in sample 53, where H units were measured to be 7.2% in the enzyme lignin and 4.5% in the protease treated lignin – a reduction of 37.5%. However, despite the reduction in observed H units, all four of these samples still had H unit measurements much higher than the population average and maintained their outlier status. This indicates that these samples may indeed have a high level of authentic H units, though not quite as high as the enzyme lignin measurements would suggest. No spatial or environmental factors impacting these 10 samples were identified. These samples will be further analyzed to investigate the presence of high H units.

Figure SI 2 – Summary of H unit levels in four selected samples before and after protease treatment.

**Figure SI 3 - Lignin phenotype correlations**

A multivariate analysis was performed in JMP to elucidate associations between the twelve phenotypes quantified by HSQC NMR. The lower triangular matrix depicts a bivariate XY plot of the two intersecting phenotypes along the diagonal. The upper triangle matrix displays the numerical Pearson correlation coefficient, along with asterisk(s) to indicate the *p*-value associated with correlation probability (** = *p*-value <0.01; * = *p*-value < 0.05; no asterisk = *p*-value >0.05).


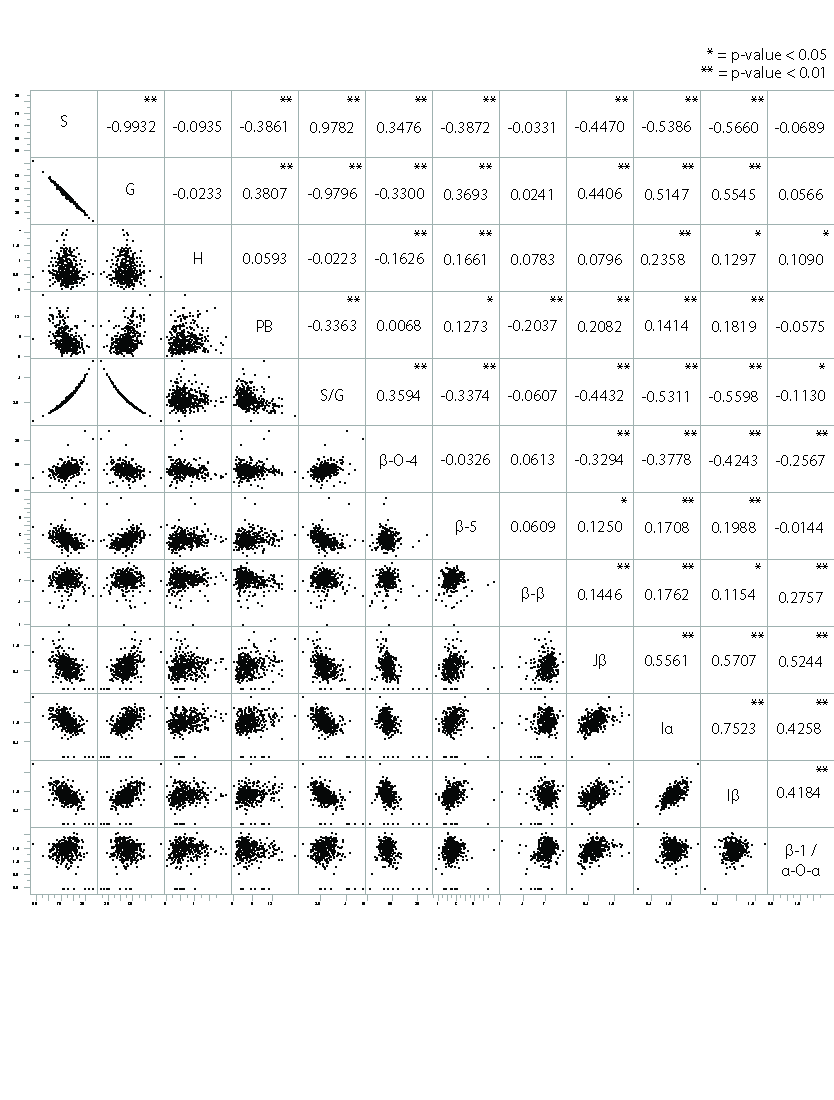


**Figure SI 4 – Gene Ontology Enrichment Results**

The candidate genes identified by GWAS analyses were examined for gene ontology (GO) terms. A GO enrichment analysis was conducted for each significance level of GWAS results (log6, log7, log8). The enrichment of cell wall (CW) related terms (i.e., number of CW related genes compared to total number of genes identified) increases with significance level. This trend indicates that the GWAS analyses are discriminately identifying CW related causal genes, as opposed to identifying random SNPs from the genome. Enrichment and p-values were obtained via hypergeometric test to compare the significance of the overlap between the number of CW genes detected by the analysis and the theoretical number obtained by random sampling.


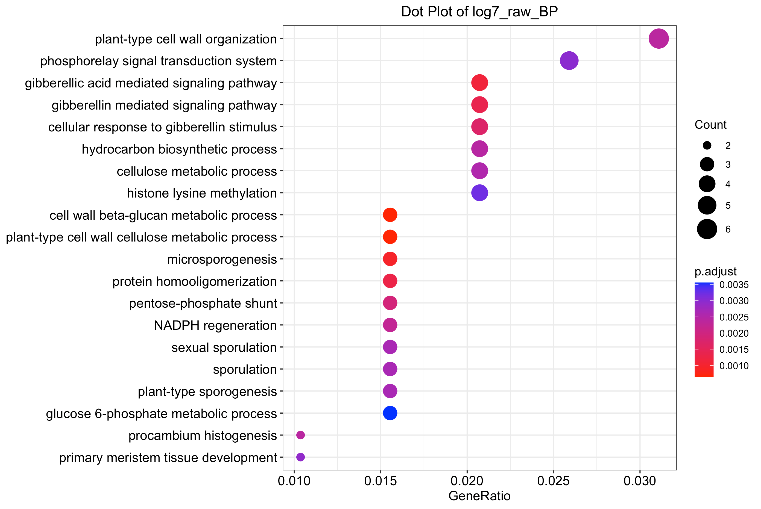

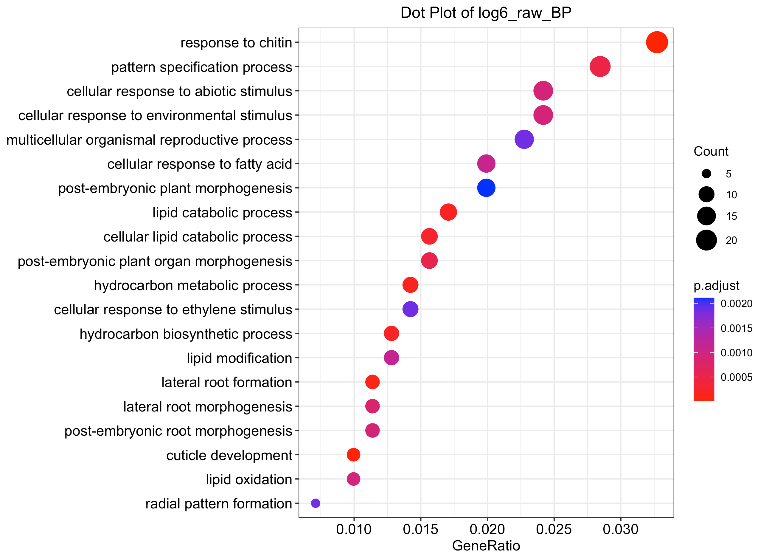

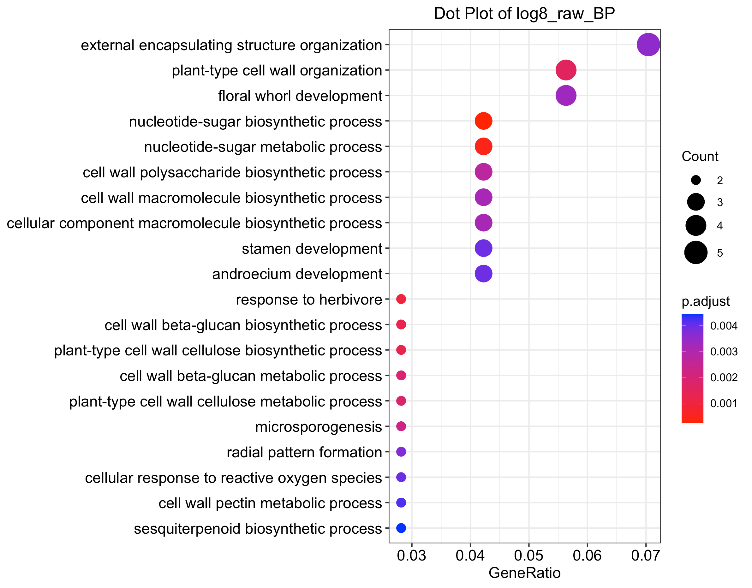


**Table SI 2 – 32 (of 756 total) genes identified by GWAS at the -log_10_(*p*-value)=6 threshold previously associated with cell wall biosynthesis**

| H | AT3G21240 | 4CL2 | 4-coumarate:CoA ligase 2 | encodes an isoform of 4-coumarate:CoA ligase (4CL), which is involved in the last step of the general phenylpropanoid pathway. The catalytic efficiency was in the following (descending) order: p-coumaric acid, caffeic acid, ferulic acid, 5-OH-ferulic acid and cinnamic acid. At4CL2 was unable to use sinapic acid as substrate. |
| --- | --- | --- | --- | --- |
| H | AT4G20050 | QRT3 | Pectin lyase-like superfamily protein | Encodes a polygalacturonase that plays a direct role in degrading the pollen mother cell wall during microspore development. |
| H | AT1G08200 | AXS2 | UDP-D-apiose/UDP-D-xylose synthase 2 | Encodes a putative UDP-D-apiose/UPD-D-xylose synthetase. |
| H | AT1G27440 | ATGUT1 | Exostosin family protein | - |
| B-5 | AT1G52760 | LysoPL2 | lysophospholipase 2 | Encodes a lysophospholipase 2 (LysoPL2). Involved in tolerance to cadmium-induced oxidative stress. Binds Acyl-CoA-binding protein 2 (ACBP2). |
| H | AT3G21240 | 4CL2 | 4-coumarate:CoA ligase 2 | encodes an isoform of 4-coumarate:CoA ligase (4CL), which is involved in the last step of the general phenylpropanoid pathway. The catalytic efficiency was in the following (descending) order: p-coumaric acid, caffeic acid, ferulic acid, 5-OH-ferulic acid and cinnamic acid. At4CL2 was unable to use sinapic acid as substrate. |
| H | AT1G15950 | ATCCR1 | cinnamoyl coa reductase 1 | Encodes a cinnamoyl CoA reductase. Involved in lignin biosynthesis. |
| I_B | AT1G78240 | OSU1 | S-adenosyl-L-methionine-dependent methyltransferases superfamily protein | Encodes TSD2 (TUMOROUS SHOOT DEVELOPMENT2), a putative methyltransferase with an essential role in cell adhesion and coordinated plant development. |
| H | AT5G14650 | - | Pectin lyase-like superfamily protein | - |
| H | AT3G02210 | COBL1 | COBRA-like protein 1 precursor | - |
| H | AT3G14310 | ATPME3 | pectin methylesterase 3 | encodes a pectin methylesterase, targeted by a cellulose binding protein (CBP) from the parasitic nematode Heterodera schachtii during parasitism. |
| H | AT4G04920 | SFR6 | sensitive to freezing 6 | Encodes a nuclear targeted protein that plays a role in the CBF pathway -downstream of CBF translation. Mutants have impaired cold responses, reduced levels of cold induced RNA transcripts, are sensitive to osmotic stress. |
| S_G | AT5G03170 | ATFLA11 | FASCICLIN-like arabinogalactan-protein 11 | Encodes FLA11, a member of fasciclin-like arabinogalactan proteins (FLAs) containing a cell adhesion fasciclin (FAS) domain. Mutations result in altered stem biomechanics with reduced tensile strength and reduced tensile modulus of elasticity, as well as altered cell wall architecture and composition, with increased cellulose microfibril angle, reduced arabinose, galactose and cellulose content. |
| H | AT2G37130 | - | Peroxidase superfamily protein | - |
| PB | AT4G24910 | - | Protein of unknown function (DUF579) | - |
| PB | AT3G03050 | ATCSLD3 | cellulose synthase-like D3 | encodes a cellulose synthase like protein. mutations initiate root hairs that rupture at their tip soon after initiation. is required for the synthesis of a noncellulosic wall polysaccharide. |
| H | AT3G28150 | TBL22 | TRICHOME BIREFRINGENCE-LIKE 22 | Encodes a member of the TBL (TRICHOME BIREFRINGENCE-LIKE) gene family containing a plant-specific DUF231 (domain of unknown function) domain. A putative xyloglucan O-acetyltransferase. TBL gene family has 46 members, two of which (TBR/AT5G06700 and TBL3/AT5G01360) have been shown to be involved in the synthesis and deposition of secondary wall cellulose, presumably by influencing the esterification state of pectic polymers. A nomenclature for this gene family has been proposed (Volker Bischoff & Wolf Scheible, 2010, personal communication). |
| H | AT5G51640 | TBL17 | Plant protein of unknown function (DUF828) | Encodes leaf-senescence-related protein. A member of the TBL (TRICHOME BIREFRINGENCE-LIKE) gene family containing a plant-specific DUF231 (domain of unknown function) domain. TBL gene family has 46 members, two of which (TBR/AT5G06700 and TBL3/AT5G01360) have been shown to be involved in the synthesis and deposition of secondary wall cellulose, presumably by influencing the esterification state of pectic polymers. A nomenclature for this gene family has been proposed (Volker Bischoff & Wolf Scheible, 2010, personal communication). |
| PB | AT3G58790 | GAUT15 | galacturonosyltransferase 15 | Encodes a protein with putative galacturonosyltransferase activity. |
| H | AT5G17200 | - | Pectin lyase-like superfamily protein | - |
| H | AT5G44670 | - | Domain of unknown function (DUF23) | - |
| H | AT2G33100 | ATCSLD1 | cellulose synthase-like D1 | encodes a gene similar to cellulose synthase |
| H | AT5G17200 | - | Pectin lyase-like superfamily protein | - |
| H | AT4G33230 | - | Plant invertase/pectin methylesterase inhibitor superfamily | - |
| B-O-4 | AT1G78060 | - | Glycosyl hydrolase family protein | - |
| H | AT1G68560 | ATXYL1 | alpha-xylosidase 1 | Encodes a bifunctional alpha-l-arabinofuranosidase/beta-d-xylosidase that belongs to family 3 of glycoside hydrolases. |
| B-O-4 | AT3G10720 | - | Plant invertase/pectin methylesterase inhibitor superfamily | - |
| H | AT4G25810 | XTH23 | xyloglucan endotransglycosylase 6 | xyloglucan endotransglycosylase-related protein (XTR6) |
| H | AT5G20680 | TBL16 | TRICHOME BIREFRINGENCE-LIKE 16 | Encodes a member of the TBL (TRICHOME BIREFRINGENCE-LIKE) gene family containing a plant-specific DUF231 (domain of unknown function) domain. TBL gene family has 46 members, two of which (TBR/AT5G06700 and TBL3/AT5G01360) have been shown to be involved in the synthesis and deposition of secondary wall cellulose, presumably by influencing the esterification state of pectic polymers. A nomenclature for this gene family has been proposed (Volker Bischoff & Wolf Scheible, 2010, personal communication). |
| PB | AT3G48950 | - | Pectin lyase-like superfamily protein | - |
| H | AT2G38080 | ATLMCO4 | Laccase/Diphenol oxidase family protein | LAC4 appears to have laccase activity based on enzyme assays performed using lac4 mutants. These mutants also have reduced levels of lignin. LAC4 is expressed in vascular bundles and fibers and likely contributes to lignin biosynthesis, and hence cell wall biosynthesis, there. lac4/irx12 mutants have a mild irregular xylem phenotype. |
| H | AT1G15950 | ATCCR1 | cinnamoyl coa reductase 1 | Encodes a cinnamoyl CoA reductase. Involved in lignin biosynthesis. |
|  |  |  |  |  |

**References**

Kim, H., Padmakshan, D., Li, Y., Rencoret, J., Hatfield, R. D., & Ralph, J. (2017). Characterization and elimination of undesirable protein residues in plant cell wall materials for enhancing lignin analysis by solution-state nuclear magnetic resonance spectroscopy. *Biomacromolecules, 18*(12), 4184-4195.
